# Molecular Origins of Transcriptional Heterogeneity in Diazotrophic Klebsiella oxytoca

**DOI:** 10.1128/msystems.00596-22

**Published:** 2022-09-08

**Authors:** Tufail Bashir, Rowan D. Brackston, Christopher Waite, Ioly Kotta-Loizou, Matthew R. Carey, Christoph Engl, Martin Buck, Jörg Schumacher

**Affiliations:** a Faculty of Natural Sciences, Department of Life Sciences, Imperial College London, London, United Kingdom; b School of Biological & Chemical Sciences, Queen Mary University of London, London, United Kingdom; University of California San Diego

**Keywords:** mathematical modeling, nitrogen fixation, transcriptional regulation

## Abstract

Phenotypic heterogeneity in clonal bacterial batch cultures has been shown for a range of bacterial systems; however, the molecular origins of such heterogeneity and its magnitude are not well understood. Under conditions of extreme low-nitrogen stress in the model diazotroph Klebsiella oxytoca, we found remarkably high heterogeneity of *nifHDK* gene expression, which codes for the structural genes of nitrogenase, one key enzyme of the global nitrogen cycle. This heterogeneity limited the bulk observed nitrogen-fixing capacity of the population. Using dual-probe, single-cell RNA fluorescent *in situ* hybridization, we correlated *nifHDK* expression with that of *nifLA* and *glnK*-*amtB*, which code for the main upstream regulatory components. Through stochastic transcription models and mutual information analysis, we revealed likely molecular origins for heterogeneity in nitrogenase expression. In the wild type and regulatory variants, we found that *nifHDK* transcription was inherently bursty, but we established that noise propagation through signaling was also significant. The regulatory gene *glnK* had the highest discernible effect on *nifHDK* variance, while noise from factors outside the regulatory pathway were negligible. Understanding the basis of inherent heterogeneity of nitrogenase expression and its origins can inform biotechnology strategies seeking to enhance biological nitrogen fixation. Finally, we speculate on potential benefits of diazotrophic heterogeneity in natural soil environments.

**IMPORTANCE** Nitrogen is an essential micronutrient for both plant and animal life and naturally exists in both reactive and inert chemical forms. Modern agriculture is heavily reliant on nitrogen that has been “fixed” into a reactive form via the energetically expensive Haber-Bosch process, with significant environmental consequences. Nitrogen-fixing bacteria provide an alternative source of fixed nitrogen for use in both biotechnological and agricultural settings, but this relies on a firm understanding of how the fixation process is regulated within individual bacterial cells. We examined the cell-to-cell variability in the nitrogen-fixing behavior of Klebsiella oxytoca, a free-living bacterium. The significance of our research is in identifying not only the presence of marked variability but also the specific mechanisms that give rise to it. This understanding gives insight into both the evolutionary advantages of variable behavior as well as strategies for biotechnological applications.

## INTRODUCTION

Cell-to-cell variability in gene expression has been recognized across many different cell types, and it has been attributed to a range of causes (1, 2). In bacteria, such variability has been suggested to underpin phenotypic heterogeneity between otherwise-genetically identical cells. Understanding the origins of cell-to-cell variability is important for medical and biotechnological applications and can lead to inferences about its evolutionary basis and the benefits in the cells' ancestral natural environments.

A key source of phenotypic heterogeneity is known to be the inherent stochasticity of transcription (1, 3, 4). Such stochasticity is common to all chemical reaction systems involving small numbers of molecules, of which transcription is a key example. Transcription can occur in bursts (5–8), characterized as short periods of intense transcriptional activity, resulting in increased heterogeneity. Together, inherent stochasticity and burstiness lead to intrinsic noise, which may be a fundamental property of transcription of a given gene. However, in addition to intrinsic noise, other sources of noise external to a particular gene (extrinsic noise) may also be at play but are often hard to determine within native gene expression settings. These additional contributions to heterogeneity have been observed by simultaneously measuring expression of two or more copies of the same gene at the single-cell level (9–11). Correlations in expression of these two gene copies reflect perturbations that simultaneously affect both.

Transcriptional noise contributions can be inferred via modeling. Intrinsic noise and bursty transcription have been modeled by the so-called telegraph process, which describes the stochastic changes in gene transcriptional activity associated with bursty transcription and predicts the probability distribution of mRNA copy numbers (12–15). Together with studies at the single-cell level, this model and its variants have been used to infer transcriptional properties (16–19) and provide improved analysis and understanding from experimental data. Such models can be extended to include effects of extrinsic noise (20–22) and subsequently allow quantification of contributions from various molecular sources and their attendant mechanisms to the total observed heterogeneity.

Phenotypic heterogeneity may be particularly relevant in costly microbial stress response systems, such as the nitrogen starvation response (16). In organisms such as Klebsiella oxytoca, nitrogen starvation triggers a transition to diazotrophy in which bacterial cells use atmospheric dinitrogen as nitrogen source for growth (23); this reaction is enabled by the nitrogenase enzyme. In the seminal work of Schreiber et al., significant diazotrophic heterogeneity was observed in K. oxytoca under nitrogen-limiting balanced growth conditions in a chemostat (16). This heterogeneity was attributed to noise acting downstream of the regulatory gene *glnK* and was further demonstrated to provide an advantage at the population level. Given that a native resource availability for enteric bacteria may be subject to sudden changes, in this work we studied transcriptional heterogeneity following the abrupt onset of nitrogen starvation. Using dual-probe single-cell RNA fluorescence *in situ* hybdridization (RNA-FISH), we further investigated the molecular origins of the transcriptional heterogeneity following a transition to diazotrophy.

**FIG 1:**
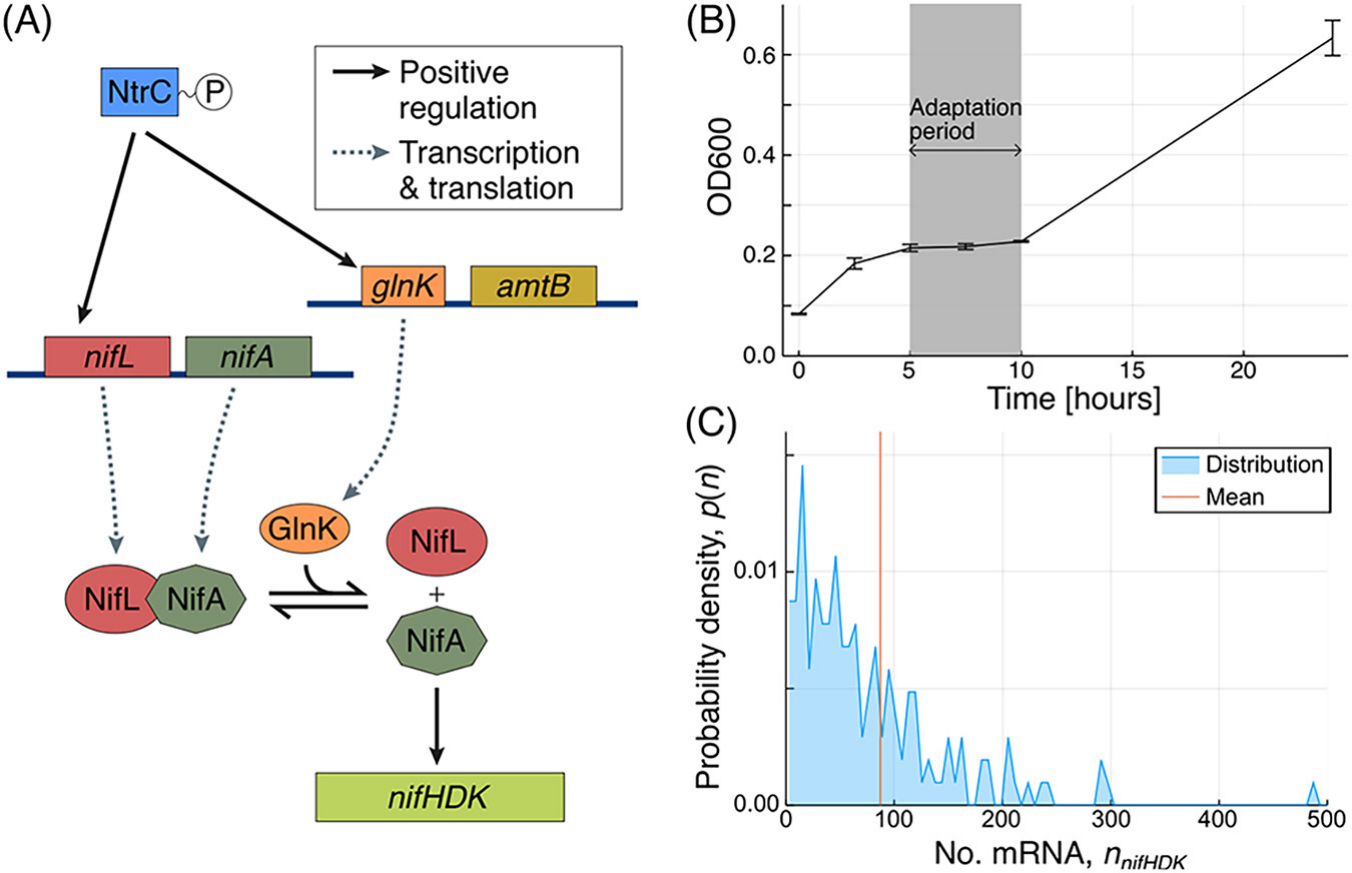
(A) Regulatory pathway governing *nifHDK* expression. Transcription of *nifHDK* is subject to a hierarchical regulatory system. (B) Population size during transition to diazotrophy in wild-type K. oxytoca. Following run-out of ammonia, cultures displayed arrested growth during the diazotrophic transition, marked in gray. Growth from 10 h onwards was achieved through nitrogen fixation. (C) Distribution of *nifHDK* transcript abundance at 8 h, as illustrated by mRNA-FISH. Adapted from reference 23 with permission from the publisher.

In K. oxytoca, expression of a functional nitrogenase involves coordinated transcription of 18 *nif* genes that are organized in 5 operons. The *nifHDK* operon encodes structural genes of nitrogenase and is the most highly expressed operon within the *nif* cluster. The core hierarchical regulatory system of *nif* expression (Fig. 1A) consists of the nitrogen regulator NtrC activating expression of *glnK-amtB* and *nifLA* operons, with NifA activating *nifHDK* gene expression when not directly inhibited by NifL (23). NtrC and NifA are bacterial enhancer binding proteins (bEBP) that activate σ^54^ RNA polymerase with a distinct ATPase-dependent activating mechanism, compared with canonical σ^70^-type RNA polymerases (24). Studies with purified components from Azotobacter vinelandii have shown that complex formation between NifL and NifA is influenced by the binding of GlnK and adenosine nucleotides to NifL, the redox status of the flavin cofactor in NifL, and the binding of 2-oxoglutarate to NifA (23, 25). Some of these regulatory features are also likely to operate for control of NifA in K. oxytoca (26). This arrangement integrates signals conducive to nitrogen fixation, i.e., a reducing environment, high energy levels, and presumably low nitrogen levels, as high 2-oxoglutarate levels indicate a low nitrogen status in the closely related Escherichia coli (27). Low nitrogen status, defined as the ratio of glutamine to 2-oxoglutarate, also increases NtrC activity and enhances its expression by triggering uridylation of P_II_ signaling proteins to allow higher overall NtrB kinase activity. Low glutamine levels affect the posttranslational uridylylation state of GlnK; however, it is unclear if the uridylation state affects GlnK function in destabilizing the NifL-NifA complex in K. oxytoca (28).

We were interested in the relative contributions of intrinsic and extrinsic noise to expression of the native single-copy *nifHDK* gene locus under conditions in which free-living wild-type K. oxytoca cells transition to use atmospheric dinitrogen as nitrogen source for growth. Using mRNA-FISH and examining the relationship between control genes at different levels in the regulatory cascade controlling nitrogenase expression, we were able to reveal the combined roles of intrinsic noise at the level of the *nifHDK* promoter and extrinsic noise arising from upstream regulation. By fitting stochastic models for transcription, these contributions were quantified, along with further details of the regulatory mechanisms. We provide evidence that heterogeneity in nitrogenase expression in wild type (WT) cells is an inherent property of the transcriptional program, and therefore we hypothesize that heterogeneity of diazotrophy could have evolved through providing advantages in natural bacterial environments.

## RESULTS

### Significant *nifHDK* mRNA heterogeneity observed in cells by RNA-FISH.

To measure the full spectrum of heterogeneity pertaining to transition into and establishment of full diazotrophy, precultures grown under nitrogen-replete aerobic conditions were transferred to nitrogen-free media under anaerobic conditions at time zero, when no *nifHDK* expression or acetylene reduction was detectable. We found that K. oxytoca (WT) populations subjected to these conditions first consumed residual ammonia and then experienced a 5-h growth arrest (Fig. 1B). This was followed by growth resumption using N_2_-derived ammonia as nitrogen source, evidenced by acetylene reduction activity profiles as a measure of bulk population nitrogenase activity in batch culture (29).

To investigate and verify the regulatory roles of *glnB*, *glnK*, and *nifA*, we tracked population growth and nitrogenase activity of wild-type and derivative mutant strains lacking the positive regulator GlnK (Δ*glnK*) and P_II_ (Δ*glnB*), as well as cells lacking bEBP NifA and its cotranscribed inhibitor, NifL (Δ*nifLA*).

We found that the absence of GlnB had negligible effects on either growth or nitrogen fixation, while absence of GlnK had an inhibitory effect on growth (see Fig. S1 in the supplemental material). Since the Δ*glnK* mutant had a growth defect under diazotrophic growth conditions (see Fig. S1A) and our previous work at an early time point (3 h post–NH_4_ run-out) had illustrated that its nitrogenase activity is significantly reduced (30), cells for RNA-FISH were harvested when they showed similar levels of nitrogenase activity across all bacterial strains, i.e., at 9.5, 14.5, and 19 h post–NH_4_ run-out (see Fig. S1B). In contrast, the Δ*nifLA* mutant exhibited both very much slower growth and a very greatly reduced acetylene reduction compared with the WT (see Fig. S1), consistent with previous findings (23).

10.1128/msystems.00596-22.1 FIG S1 Growth and acetylene reduction assay results with K. oxytoca strains at sampling times for RNA-FISH experiments. (A) OD_600_ at time points 0, 9.5, 14.5, and 19.5 h. Error bars represent standard deviations from the means (*n* = 6). (B) Ethylene produced, in nanomoles per hour per milliliter and per OD_600_ unit. Error bars represent standard deviations from the means (*n* = 2). Download FIG S1, TIF file, 0.09 MB. Copyright © 2022 Bashir et al. 2022 Bashir et al. https://creativecommons.org/licenses/by/4.0/ This content is distributed under the terms of the Creative Commons Attribution 4.0 International license.

We established use of mRNA-FISH (31) in K. oxytoca to estimate mRNA levels for the *nifHDK* operon, which encodes the Fe protein component of nitrogenase. Results indicated significant variation in *nifHDK* transcript abundance between cells (Fig. 1C). As is common in gene expression data, the distribution was decidedly non-Gaussian, with a bulk of cells at low expression levels and a longer tail of much higher values. Hence, the “average” cell had relatively high expression levels compared with the majority, and therefore bulk measurements of *nifHDK* gene expression did not represent those of a typical cell.

### Mutual information analysis confirmed direct propagation of transcriptional noise to *nifHDK* via GlnK.

**FIG 2:**
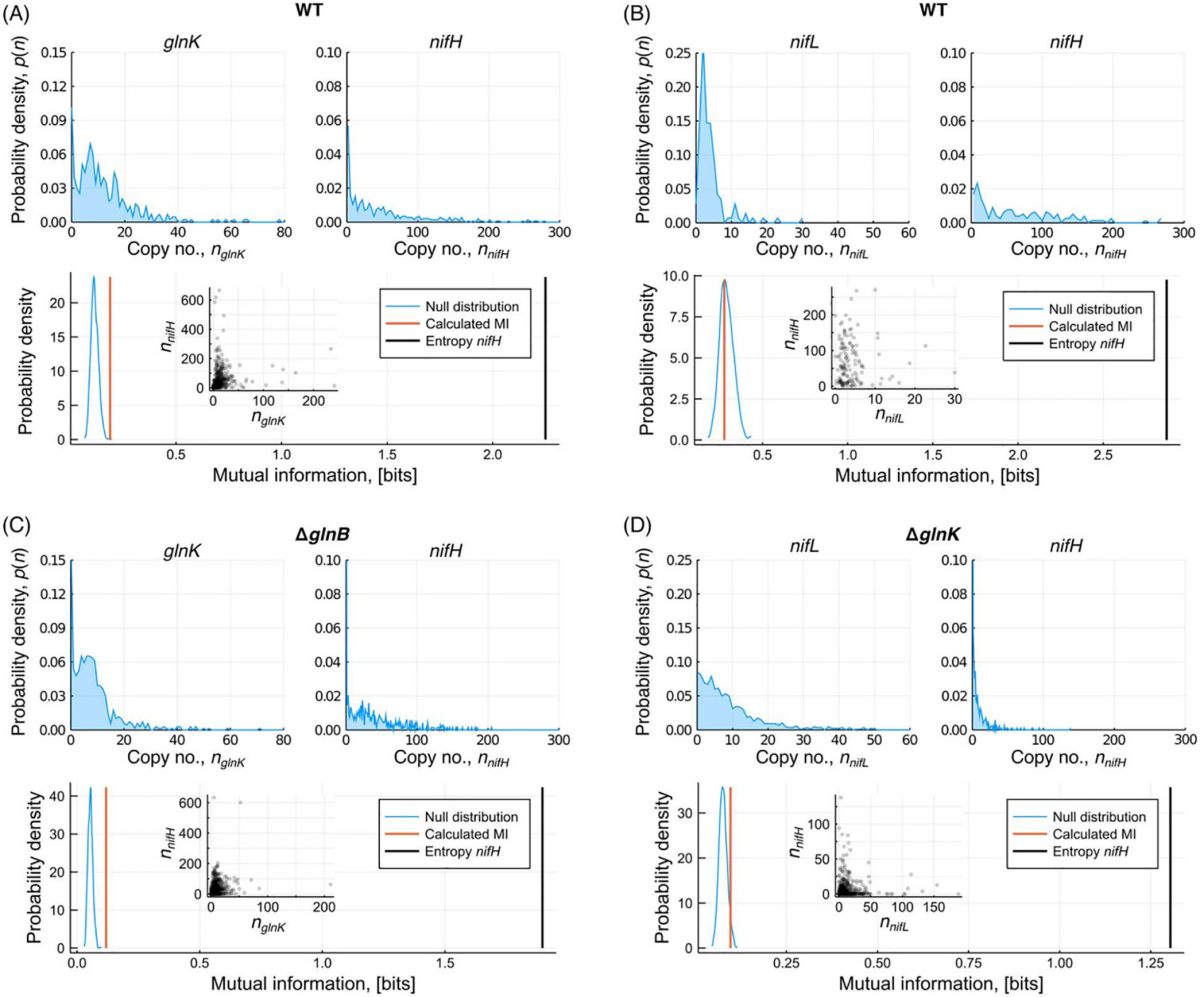
Mutual information analysis based on dual-probe measurements from biological replicate 1. For each case, the marginal distributions of the two mRNA abundances are shown, in addition to the calculated mutual information and the total entropy in *nifHDK* abundance. Mutual information was compared to a null distribution obtained by randomly shuffling the *nifHDK* data 100,000 times, thereby providing a *P* value for each pair. These values for the displayed data and a further biological replicate are displayed in Table 1. (A) WT cells in which *glnK* and *nifH* were simultaneously measured, indicating significant MI. (B) WT cells in which *nifL* and *nifH* were simultaneously measured, indicating no significant MI. (C) Cells from the Δ*glnB* mutant in which *glnK* and *nifH* were simultaneously measured, indicating significant MI. (D) Cells from the Δ*glnK* mutant in which *nifL* and *nifH* were simultaneously measured, indicating no significant MI.

Given significant heterogeneity in *nifHDK* gene expression (Fig. 1C and 2), we sought to establish whether extrinsic noise is present and if this noise arises from the regulatory pathway or from elsewhere. We grew WT cells and Δ*glnK* and Δ*glnB* mutants to levels where different bacterial cultures displayed equivalent cumulative levels of nitrogenase activity (as described in Materials and Methods). Two sets of probes were used to measure *nifHDK* expression and simultaneously either *glnK* or *nifLA* expression in individual cells to monitor variability across these paired genes. Expression covariance between two genes can occur either because one gene has a direct (and relatively quick) influence on the other, or because both genes are simultaneously affected by another source of variability. This could be a shared and rather specific upstream control protein or more “global” factors, such as RNA polymerase or sigma factor abundance, which may influence many genes simultaneously and should therefore be evident as significant covariance between any expressed pair of genes.

We used mutual information (MI) as a suitable metric for quantifying covariance in gene expression. MI is commonly used to analyze single-cell data in situations where relationships are complex, nonlinear, and unknown, as no assumptions or prior knowledge are required (32, 33). Here, we computed the MI between each gene pair and a null distribution of MI values from which a corresponding significance level could be computed (see Materials and Methods). The MI can be compared further to the entropy of the *nifHDK* expression level distribution, to compare the MI with the total information content.

The results (Fig. 2 and Table 1) demonstrated modest MI between *glnK* and *nifHDK* for both the WT (Fig. 2A) and Δ*glnB* (Fig. 2C) strains, but there was no statistically significant measure of MI between *nifLA* and *nifHDK* (Fig. 2B). Undetectable MI between *nifLA* and *nifHDK* suggests that global sources of extrinsic noise are not significant for these two operons. By extension, one can deduce that such global factors are therefore unlikely to be generally significant for σ^54^-dependent genes, since by definition global factors influence many genes simultaneously. The measurable low-level MI between *glnK* and *nifHDK* was therefore suggestive of a direct propagation of noise from *glnK* to *nifHDK*. Thus, stochastic variability of *glnK* expression acts to increase the level of variability in *nifHDK* expression; cells with high *glnK* mRNA levels at a given time are also likely to have higher *nifHDK* mRNA levels. Heterogeneity in *nifHDK* expression is therefore generated at multiple levels in the regulatory cascade, suggesting that it is a fundamental property of the regulatory system.

**TABLE 1:** Mutual information with *nifHDK* transcript abundance^*a*^

Genetic strain	Gene	Biological replicate no.	Mutual information (bits)	Entropy of *nifHDK* (bits)	Significance level
WT	*glnK*	1	0.187939	2.249837	0.00016
WT	*glnK*	2	0.340274	2.444729	0.00035
WT	*nifL*	1	0.275314	2.872011	0.61815
WT	*nifL*	2	0.31771	2.752161	0.47135
Δ*glnB*	*glnK*	1	0.118429	1.897344	0.00000
Δ*glnB*	*glnK*	2	0.071995	1.915492	0.00744
Δ*glnK*	*nifL*	1	0.066759	0.690746	0.27533
Δ*glnK*	*nifL*	2	0.096455	1.303811	0.04686

a The entropy of *nifHDK* abundance provided a measure of the total variability, while the mutual information was the amount of variability that could be directly explained by the abundance of the other gene. Smaller significance levels indicate a stronger statistical indication of nonzero mutual information.

### Stochastic models incorporating extrinsic noise.

**FIG 3:**
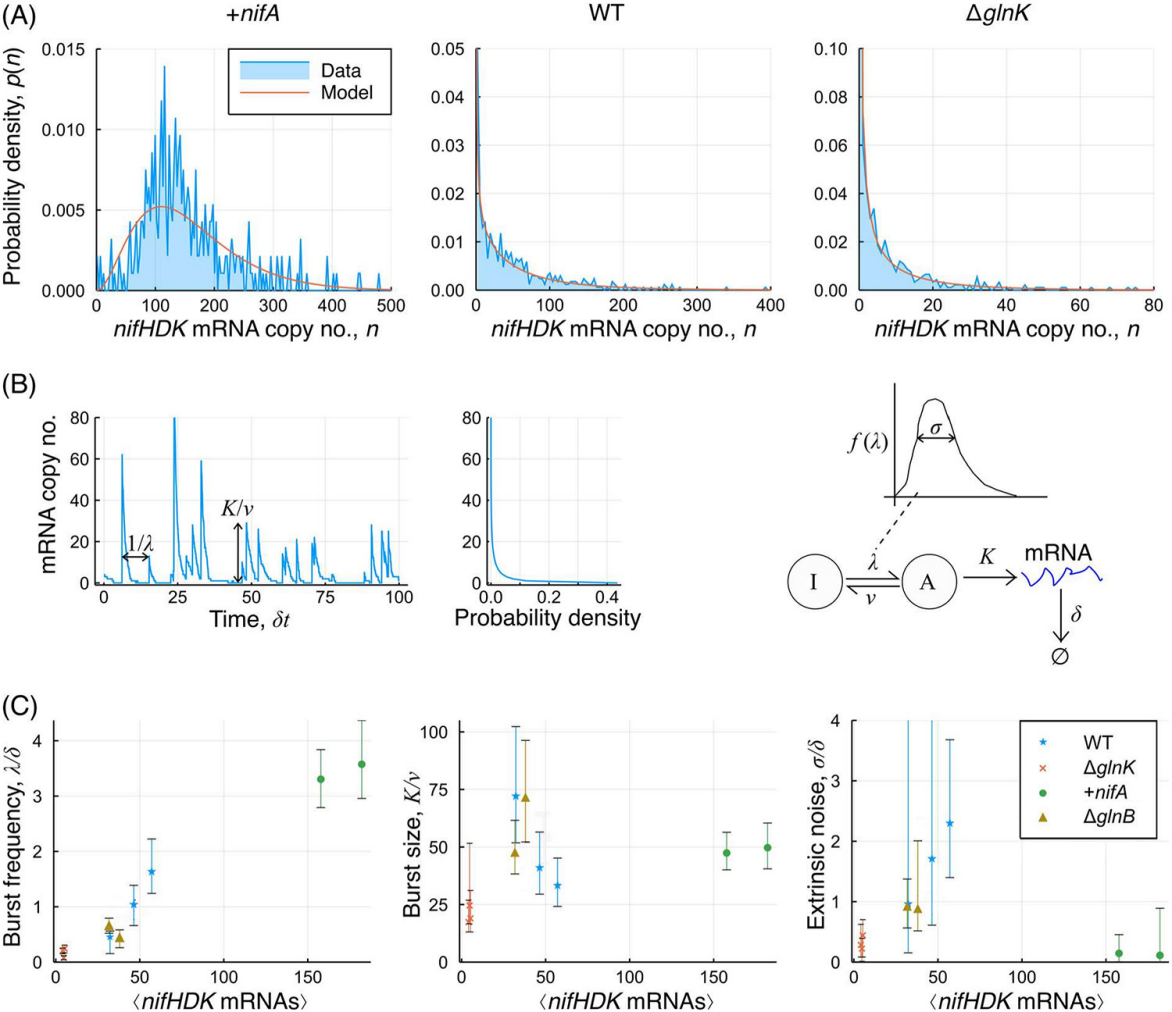
Stochastic modeling of bursty transcription incorporating extrinsic noise. (A) *nifHDK* mRNA copy number distributions for each of the +*nifA*, WT, and Δ*glnK* strains. WT and Δ*glnK* data are from replicate 1 as displayed in Fig. 2. (B) Schematic of bursty transcription and its relation to the stochastic model. Extrinsic noise here is catered for by considering the burst frequency to be variable between cells. (C) Variation of the model parameters between mutants, plotted as a function of the mean expression level. Error bars denote 95% Bayesian credible intervals, obtained from the posterior distributions. Data are plotted for biological replicates 1 and 2.

Next, we sought to understand the minimum level of variability that might be observed if the direct regulatory role of GlnK were bypassed. We measured the expression levels in Δ*nifLA* in which *nifA* was overexpressed ectopically (+*nifA*), making *nifHDK* expression independent of GlnK. Figure 3A displays a distribution for the +*nifA* mutant, showing significant remaining sources of heterogeneity. Given that fluctuations in GlnK are no longer anticipated to have an effect on *nifHDK* expression heterogeneity and that global sources of extrinsic noise were found to be minimal, *nifHDK* transcription heterogeneity must have been due to intrinsic noise at the level of the *nifHDK* promoter. Such noise is widely attributed to bursty transcription, in which the promoter is intermittently active and inactive (5, 6). The sources of this intermittent activity may be related to transcription factor binding and unbinding (34) or to mechanical supercoiling effects (35, 36). Regardless of the mechanism, similar levels of intrinsic noise may also be relevant for the other genetic variants (WT, Δ*glnK*, etc.).

Since both intrinsic and extrinsic sources of noise may be generally relevant, we sought to develop stochastic models for transcription that could incorporate both these effects (22). When considering *nifHDK* transcription to be intrinsically bursty, as shown schematically in Fig. 3B, this can be modeled by the telegraph model (13, 15), where the promoter undergoes rapid activation and deactivation at rates λ and ν, respectively. When active, transcription occurs at rate *K*, while the mRNA is degraded in a first-order degradation process with rate *δ*. Under particular parameter ranges, this process results in a negative binomial distribution for the transcript copy number (22, 37), parameterized by the normalized burst frequency λ/δ and mean burst size *K*/ν. In this context, we took the view that extrinsic noise arising from *glnK* variability acts as a variation between cells in burst frequency. This led to a model that is additionally parametrized by the normalized frequency variation σ/δ (see Materials and Methods).

For each of the three exemplar distributions shown in Fig. 3A, a comparison is given with the model following the parameter fitting process. The model can provide a good fit to the data in each case, enabling us to draw meaning from the model parameters, inferred for a number of genetic variants and biological replicates, as displayed in Fig. 3C. We observed a clear relationship between mean expression and burst frequency, which was contrasted by the very limited correlation between mean expression and burst size. Cells such as Δ*glnK* have a very low burst frequency, yet their burst size is within a factor of 2 of that for the most highly expressing +*nifA* strain. Finally, it was clear that extrinsic noise was lowest in both the Δ*glnK* and +*nifA* strains. This supports the findings from the MI analysis that extrinsic noise arises from *glnK*, as such noise was reduced when *glnK* was either absent or bypassed.

**FIG 4:**
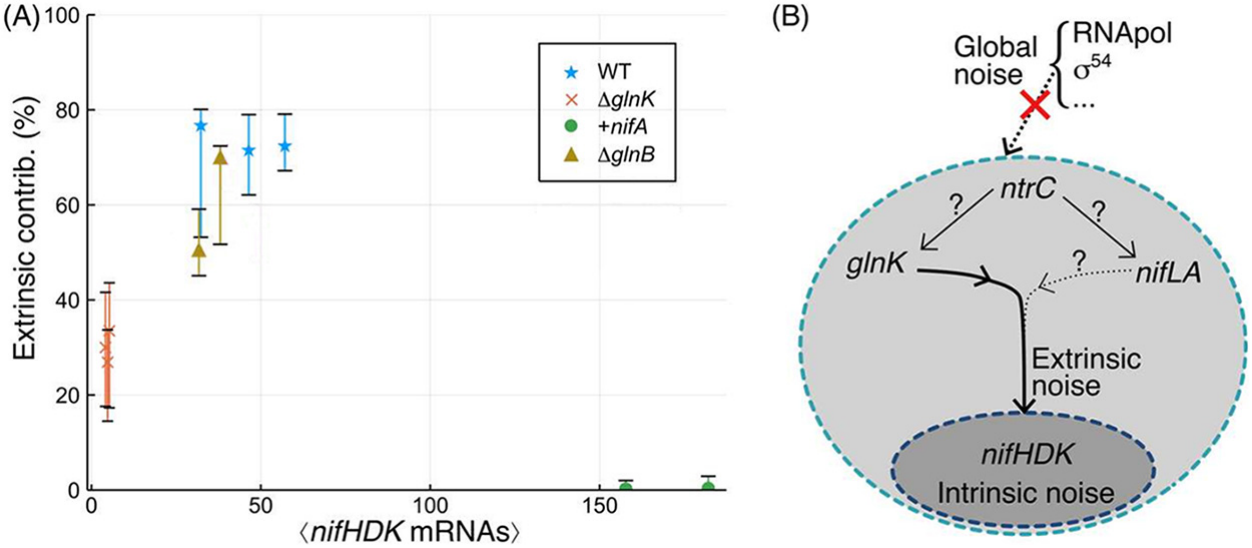
(A) Calculated contributions of extrinsic noise to the variance in *nifHDK* transcript abundance. Contributions are calculated for 4,000 parameter triplets sampled from the posterior distributions, thereby providing the most probable value and 68% credible intervals. (B) Schematic showing propagation of noise through the signaling cascade. Only a contribution from *glnK* was supported directly by the experimental data, although contributions from *nifLA* and from *ntrC* could not be excluded. Global noise sources were determined to be undetectable.

Based on these model fits, we further calculated the contributions of intrinsic and extrinsic noise to the total variance in *nifHDK* expression (21, 38) (see Materials and Methods), as shown in Fig. 4A. For Δ*glnK*, the results demonstrated that a significant contribution from extrinsic noise of ≈30% remained, indicating that some variability in burst frequency may have arisen from noise sources other than the cell-to-cell variation in GlnK activity. The extrinsic noise contribution increased to ≈70% in WT and Δ*glnB*, in which noise propagation from *glnK* is known to be present, but was almost zero in the +*nifA* mutant, in which the nitrogen regulatory pathway via GlnK is uncoupled.

## DISCUSSION

Bacteria can be subject to a multitude of stresses and respond in a multitude of ways. While the average behavior of a clonal population is often of interest, the degree of variability between cells is also important. Such knowledge may provide both mechanistic biochemical insights as well as an indication of typical survival strategies that leverage this variability. Here, we examined the nitrogen starvation response in a model diazotroph, determining behavior at the population level and expression of several genes in the regulatory cascade at single-cell resolution.

From bulk measurements, our data extended the existing understanding of the *nif* regulatory network in K. oxytoca. We found that a Δ*glnB* strain behaved similar to WT in terms of both growth and noise propagation, consistent with GlnK being able to compensate for loss of GlnB. GlnB and GlnK were shown to have redundant functionalities in regulating nitrogen assimilation genes in E. coli, but this redundancy was not fully extended to *nifHDK* gene regulation in K. oxytoca (39). Further, GlnB and GlnK posttranslational uridylylation plays an important role in regulating nitrogen assimilation genes, but GlnK uridylylation appears not to be required for *nifHDK* gene regulation *in vivo* (10). Our Δ*glnK* strain showed clearly discernible growth but similar acetylene reduction to that of the WT at late time points post–NH_4_ run-out (see Fig. S1 in the supplemental material), compared to earlier time points (30). Since our data on *nifHDK* mRNA copy numbers as quantified by RNA-FISH did not support equal NifHDK expression in Δ*glnK* and WT, the comparable nitrogenase activity may have been due to NifH protein expression and/or stability. Alternatively, increased N_2_ assimilation rates may compensate for low nitrogenase levels to help diazotrophic growth of cells lacking GlnK, or possibly the *glnB*-encoded P_II_ can partially substitute for GlnK. Nevertheless, growth of the Δ*glnK* strain remained defective despite the high nitrogenase activity, potentially due to pleotropic effects on nitrogen regulation, assimilation, and metabolism, such as the AmtB ammonia transporter inhibition normally mediated by GlnK.

It has been shown that GlnK plays a role in dissociating the repressed NifL-NifA complex in E. coli, an activity also inferred from *in vivo* transcription assays in K. oxytoca (39, 40). In contrast, the NifL-NifA complex of the diazotroph Azotobacter vinelandii is not affected by the absence of *glnK* in the heterologous host E. coli, as evidenced in *in vitro* transcription assays from the *nifH* promoter (41). Similarly, we found nonzero expression of *nifHDK* in native K. oxytoca (Δ*glnK)*, indicating that GlnK is not strictly required for the repressive dissociation of the NifL-NifA complex. It has been shown for A. vinelandii *in vitro* that NifA dissociates from NifL in the presence of 2-oxoglutarate and, in the closely related E. coli, 2-oxoglutarate concentrations are highly elevated under nitrogen starvation (27). Consistent with these findings, we propose that in K. oxytoca 2-oxoglutarate is sufficient to dissociate NifL-NifA *in vivo*, at least under the relatively rapid nitrogen depletion conditions used here. Given that metabolic changes generally occur on a much faster time scale (seconds) than regulatory mechanisms involving gene expression (many minutes), bypassing any strict *glnK* requirement under transient nitrogen starvation conditions may represent an evolved regulatory shortcut. Additionally, in K. oxytoca, GlnB when overexpressed can functionally compensate for a deletion of *glnK* (38), and because *glnB*, unlike *glnK*, is constitutively expressed, such a compensatory mechanism of *glnK* by *glnB* could explain the reduced contribution of extrinsic noise. Further, 2-oxoglutarate and adenosine nucleotides, potentially involved in GlnK releasing the NifL-NifA complex in K. oxytoca under conditions of nitrogen deprivation (41), may alternatively bind to GlnB, should this P_II_ protein act as a substitute for GlnK and lead to NifA dissociation from NifL. Finally, readily detectable low-level *nifHDK* expression did not occur in the Δ*nifLA* strain (see Fig. S2). This was consistent with a control scheme in which NifA is essential while GlnK is not. Interestingly, in diazotrophic cyanobacteria, 2-oxoglutarate is the major signal triggering differentiation into diazotrophy, although the nitrogen regulatory pathways are distinct in these non-proteobacteria (42).

10.1128/msystems.00596-22.2 FIG S2 Expression of *nifHDK* operon at low and zero levels for quantification of mRNA. (A and B) DAPI staining (A) and *nifHDK* mRNA-FISH probe signal (B). (i) M5a1 WT strain grown for 4 to 5 h with 0.5 mM ammonia; (ii) Δ*nifLA* strain grown for 4 to 5 h with 10 mM ammonia. Download FIG S2, TIF file, 1.8 MB. Copyright © 2022 Bashir et al. 2022 Bashir et al. https://creativecommons.org/licenses/by/4.0/ This content is distributed under the terms of the Creative Commons Attribution 4.0 International license.

By examining the mutual information between gene pairs, we can infer how noise is propagated through the regulatory cascade. MI was generally small compared with *nifHDK* entropy and was below the level of statistical significance between *nifLA* and *nifHDK*, suggesting that global sources of extrinsic noise were negligible. In other genetic systems, the source of variability in transcription has been attributed to variability in sigma factor or RNA polymerase abundance (1, 9, 43); our results suggest that this was not the case here. We cannot exclude that fluctuations do propagate from NifLA, since the differences in mRNA and protein lifetimes can act to reduce correlations between transcript and protein abundance (43). Fluctuations could also propagate from the master regulator NtrC, but any sources of noise above this level must be small, and therefore all variability in *nifHDK* expression must arise from within, and not outside, the regulatory network (Fig. 4B).

Unlike for *nifLA*, a small but statistically significant MI was observed between *glnK* and *nifHDK*. This indicated that variability in *glnK* expression must propagate down to *nifHDK*. We therefore have a direct detection of extrinsic noise from within the regulatory pathway acting on *nifHDK* transcription.

While the MI analysis evidenced the nonzero contribution of extrinsic noise to heterogeneity in *nifHDK* transcription, we further quantified this contribution through stochastic modeling. All single-cell measurements for *nifHDK* expression were consistent with a model in which transcription is inherently bursty, even in the +*nifA* mutant, in which the promoter should be constitutively active. This burstiness may result from unavoidable dissociation of the NifA bEBP and/or the RNA polymerase closed complex, and it acts to generate significant levels of intrinsic noise. The model also incorporates noise propagation as variability in the frequency of the transcriptional bursts. Furthermore, by examining the variation of model parameters across mutants, we were able to deduce that the average expression level was principally determined by the burst frequency rather than the burst size. This was consistent with recent observations for the phage shock protein (Psp) membrane stress response, which is also σ^54^ dependent (44), and in contrast to the existing understanding for σ^70^ promoters (45).

The model and inferred parameter variation (Fig. 3C) were consistent with initiation of bursts by binding of the NifA bEBP to its target, closed promoter complexes, and terminated by its unbinding. In the case of the +*nifA* mutant, high NifA availability enabled frequent bursts of transcription, yet the burst size was similar to that of the WT, perhaps because the average time before NifA dissociation from the promoter was independent of its abundance within the cell. This may reflect the rather slow conversion of a closed promoter complex, such as that bound by NifA, to an open promoter complex from which NifA has dissociated (46). The level of extrinsic noise in this case was very low, since NifA availability was high enough for the system to essentially be “saturated”; any variability in NifA has little impact if the burst frequency is already at a maximal value.

The modeling and quantitative analysis provided mechanistic insight into the sources of heterogeneity, demonstrating that the large variation in *nifHDK* expression was an inherent property of this system. Heterogeneity manifested as a cautiousness in fully activating *nifHDK* transcription across all cells, such that even under nitrogen-starved anaerobic conditions, many cells had few or no *nifHDK* transcripts. It is possible that this variability is either unavoidable or sufficiently benign, such that the extra regulatory effort required to suppress it is not worthwhile (47, 48). However, we observed that there was no leaky expression of nitrogenase under nitrogen-replete or aerobic conditions, suggesting that diazotrophic heterogeneity is an evolved systems characteristic that could be beneficial at the population level, perhaps as a bet-hedging strategy (49, 50). In the key previous study on heterogeneity in nitrogen fixation (16), it was determined that heterogeneity was indeed advantageous at the population level, following a downshift in nitrogen availability. Our results suggest that heterogeneity may also be advantageous in the opposite scenario, in which a starved population suddenly accesses fixed nitrogen. While nitrogen fixation is essential for growth under nitrogen-deplete conditions, the transition to diazotrophy is extremely expensive to a cell at many levels. Thus, if reactive nitrogen soon becomes available again, it is advantageous for some cells to have not fully undergone the diazotrophic transition. Activation of the nitrogen starvation response may therefore be a gamble in which the payoff depends on future conditions. In unpredictable natural environments, in contrast with controlled laboratory settings, stochastic variability in the stress response helps ensure that a subset of the population is always well placed for growth. Because resource availability for enteric bacteria is indeed highly unpredictable, such a strategy is likely advantageous.

While a bet-hedging strategy would work well for free-living bacteria, it is also worth noting that most bacteria live in biofilm communities and are associated with, for example, key biogeochemical cycles on earth (51). This includes both binary- and single-species biofilms of nitrogen-fixing organisms (52, 53). The stochastic and cautious nature of nitrogenase activation observed here may form part of a mechanism by which cells test conditions and ensure differentiation into specialized phenotypes only under the correct conditions.

The observations made here are likely applicable to many other costly stress response systems. However, particular interest in the nitrogen starvation response arises from the desire to engineer higher bulk levels of nitrogen fixation (54). In this context, the significant heterogeneity observed here naturally imposes limitations on industrial-scale use of diazotrophs (55), as well as confounding the efficient use of clonal populations of diazotrophs in the rhizosphere unless engineered to avoid variance. We hope that the results reported here will therefore motivate further work to understand and combat heterogeneity in these contexts.

## MATERIALS AND METHODS

### Bacterial strains and growth conditions.

All experiments were performed with Klebsiella oxytoca M5aI, which was obtained from Z. Yu and colleagues (56). Whole-gene knockout mutants, marked with a kanamycin resistance (*nptII*) gene, were derived from M5a1 by using Lambda red recombineering (Datsenko and Wanner method). The oligonucleotide primers used to construct knockout mutants can be found in Table S1 in the supplemental material. To generate a strain overexpressing NifA, the M5aI *nifA* gene sequence was cloned into the pSEVA424 vector (from R. Silva-Rocha and colleagues) under the control of the P*trc* promoter and a synthetic ribosome binding site (BBa_B0032, Registry of Standard Biological Parts), prior to transformation into the Δ*nifLA* mutant background by electroporation. NH_4_ run-out was used to derepress *gln* and *nif* gene expression and stimulate a reproducible transition into diazotrophic growth. Briefly, K. oxytoca strains were cultured in nitrogen-free David and Mingioli (NFDM) medium (57) (69 mM K_2_HPO_4_, 25 mM KH_2_PO_4_, 0.1 mM Na_2_MoO_4_, 90 μM FeSO_4_, 0.8 mM MgSO_4_, 2% [wt/vol] glucose) supplemented with NH_4_Cl as a nitrogen source. To ensure replete cellular N status, seed cultures were supplemented with 20 mM NH_4_Cl and grown to an optical density at 600 nm (OD_600_) of ≈2 to 3. Cells were washed and resuspended in NFDM supplemented with 0.5 mM NH_4_Cl to an OD_600_ of 0.1. Cultures were then crimp-sealed in 70-mL glass serum bottles (Wheaton) and chilled on ice while sparged with N_2_ gas for 45 min to establish a microaerobic atmosphere. Colorimetric O2xyDot sensors (OxySense) fixed inside the bottles were used to verify O_2_ concentration. Following injection of 1 mL pure, O_2_-free acetylene into the headspace, cultures were warmed to 25°C and shaken at 200 rpm for up to 24 h. We harvested cells from different bacterial strains when they were at the same growth point and exhibited the same cumulative levels of nitrogenase activity (see Fig. S1). Time points selected to achieve this uniform level of nitrogenase activity across different M5a1 bacterial strains were 14.5 h for the **Δ***glnB* and M5a1 (WT) strains and 19.5 h for the **Δ***glnK* strain.

10.1128/msystems.00596-22.5 TABLE S1 Names and sequences of the oligonucleotide primers used to construct knockout mutants. Download Table S1, DOCX file, 0.01 MB. Copyright © 2022 Bashir et al. 2022 Bashir et al. https://creativecommons.org/licenses/by/4.0/ This content is distributed under the terms of the Creative Commons Attribution 4.0 International license.

### Nitrogenase assay.

Nitrogen fixation was assessed via the acetylene reduction assay (58). For this assay, 500 μL of culture headspace was sampled via gas-tight syringe and subject to gas chromatography through a HayeSep N column (Agilent) at 90°C in N_2_ carrier gas. Acetylene and ethylene were detected by flame ionization at 300°C, and ChemStation software (Agilent) was used to integrate signal peak areas. Periodically, 15 mL of oxygen-free N_2_ gas was injected into sample bottles via gas-tight syringe prior to extraction of an equivalent volume of cell culture for analysis of OD_600_ and RNA-FISH. Accumulative nitrogenase activities are expressed as the percent acetylene consumption and ethylene production, normalized by the OD_600_ (see Fig. S1).

### RNA fluorescence *in situ* hybridization.

mRNA-FISH was performed according to a protocol described previously (31). Briefly, bacterial cells were sampled anaerobically and collected by centrifugation. Pelleted cells were fixed in a buffer containing 3.7% (vol/vol) formaldehyde prior to permeabilization in 70% (vol/vol) ethanol. Hybridization and wash steps were performed in saline and sodium citrate buffer (150 mM sodium chloride, 15 mM sodium citrate) supplemented with 40% (vol/vol) formamide. All solutions were prepared using diethyl pyrocarbonate-treated water and RNase-free plasticware. DNA probes against the *nifHDK*, *nifLA*, and *glnK*-*amtB* structural operons were designed using the Stellaris Probe Designer version 4.2; the oligonucleotide length was set at 20 nucleotides (nt), the minimal spacing length was set at 2 nt, and the masking level was set at 1 to 2. The probes were purchased prelabeled with 6-carboxytetramethylrhodamine succinimidyl ester (for *nifLA* and *glnK-amtB*) or Cy5 equivalent Quasar 670 (for *nifHDK*) from LGC Biosearch Technology. The oligonucleotide probes used for mRNA-FISH can be found in Table S2. Hybridization was performed overnight at 30°C at a final concentration of 1 μM, in buffer containing 2 mM ribonucleoside-vanadyl complex, 1 mg mL^−1^ E. coli tRNA, and 10% (wt/vol) dextran sulfate. Following multiple wash steps, chromosomal DNA was stained with 10 μg per mL 4′,6-diamidino-2-phenylindole (DAPI) for 30 min before cells were immobilized using 1% (wt/vol) agarose pads on 35-mm high μ-Dishes (ibidi) for imaging.

10.1128/msystems.00596-22.6 TABLE S2 Names and sequences of the oligonucleotide probes used for mRNA fluorescence *in situ* hybridization. Download Table S2, DOCX file, 0.02 MB. Copyright © 2022 Bashir et al. 2022 Bashir et al. https://creativecommons.org/licenses/by/4.0/ This content is distributed under the terms of the Creative Commons Attribution 4.0 International license.

For normalization purposes, we established experimental conditions that allowed us to express *nifHDK*, *nifLA*, and *glnK-amtB* operons at low and zero levels by using different strains of K. oxytoca M5a1 (see Fig. S2 to S4). These conditions were as follows: 7

Low expression of *nifHDK*: Δ*glnK* M5a1 strain was grown for 4 to 5 h with 10 mM ammonia;
Zero expression of *nifHDK*: Δ*nifLA* strain was grown for 5 to 6 h with 20 mM ammonia;
Low expression of *nifLA*: M5a1 strain was grown for 3 to 4 h with 0.5 mM ammonia;
Zero expression of *nifLA*: Δ*nifLA* strain was grown for 5 to 6 h with 20 mM ammonia;
Low expression of *glnK-amtB*: M5a1 strain was grown for 4 to 5 h with 0.5 mM ammonia;
Zero expression of *glnK-amtB*: Δ*glnG* strain was grown for 4 to 5 h with 10 mM ammonia.

### Microscopy and image analysis.

Cells were harvested for carrying out RNA-FISH and microscopy analysis as described previously (31) with slight modifications. We used 35-mm ibidi discs for imaging purposes with the help of a WF1 Zeiss Axio observer inverted microscope. Multiple fields of view were acquired for each sample, and within each field of view five *z*-slices were captured for further processing. Data stacks were converted to TIFF format using ImageJ, and cell segmentation masks from bright-field or DAPI images were generated using Schnitzcells. The protocol outlined previously (31) was then followed to detect and quantify mRNA in each cell by using the Spatzcells package in MATLAB. Fluorescent spots within cells were detected automatically by the software, and false positives were removed using a threshold chosen using the “zero” control samples described above. The probability distribution of the remaining spots was then extracted, and remaining false-positive spots were removed using the 99.9th percentile from the zero control sample of nonexpressing cells. The distribution of spots from the “low” control sample were then used to determine the characteristic intensity of a single mRNA, obtained by fitting a Gaussian distribution to the spot intensity histogram. With this normalization performed, spots could be integrated within the cell boundary determined by the masks, thereby obtaining mRNA copy numbers for each imaged cell. The number of imaged cells ranged from 200 to 1,000 for each individual sample.

### Mutual information analysis.

In order to assess the relationship between transcript abundance of two different genes, we evaluated the MI. MI is a method for evaluating statistical dependencies between two random variables from the joint and marginal probability distributions, even when the underlying relationship is complex and nonlinear. These distributions are the transcript abundance distributions obtained empirically from the data, examples of which are displayed in Fig. 2. As with any statistic intended for classification, it is important to ascertain a confidence threshold in order to rule out false positives. This is achieved by evaluating a null distribution for the value of the test statistic in the case that there is no relationship. Here, we achieved this by shuffling the data for one gene and evaluating the MI obtained in that case. By performing this shuffling and evaluation 100,000 times, a null distribution for the MI is obtained. From this null distribution, a significance level can be obtained for the measured MI.

Entropy was calculated from the transcript abundance measurements. The data were discretized into a probability distribution over the (integer) mRNA copy numbers, denoted in the following equation as p(*x*). The following formula was then applied:

$$H\left(x\right)=-\underset{x}{\sum }p(x)\log[p(x)].$$

### Stochastic modeling and parameter inference.

The stochastic model for transcription is based upon the telegraph model, in which a given gene transitions from inactive to active at rate λ and from active to inactive at rate ν. When the promoter is active, transcription occurs at rate *K*, while degradation of the mRNA occurs at rate δ independent of the promoter activity. If ν≫λ and K≫δ, the distribution of transcript abundances is the negative binomial (22):

$$p\left(n;\frac{\lambda }{\delta },\frac{K}{\nu }\right)=\text{NegBinom}\left(n;\frac{\lambda }{\delta },\frac{\nu }{\nu +K}\right)=\text{NegBinom}\left(n;r,p\right)=\left(\begin{array}{c} n+r-1\\ n \end{array}\right){\left(1-p\right)}^{r}p^{n}.$$

We additionally considered extrinsic noise arising from variation in *glnK* (and potentially other factors) to act as a variable activation rate. This was incorporated by taking the parameter λ as itself varying between cells according to a log-normal distribution with mean λ and standard deviation σ. This leads to the following compound distribution:

$$q\left(n;\frac{\lambda }{\delta },\frac{K}{\nu },\frac{\sigma }{\delta }\right)=\overset{\infty }{\underset{0}{\int }}\text{NegBinom}\left(n;r,\frac{\nu }{\nu +K}\right)\text{LogNormal}\left(r;\frac{\lambda }{\delta },\frac{\sigma }{\delta }\right)dr.$$

The model therefore gives us an expected transcript abundance distribution in terms of three parameter ratios: λ/δ, the average normalized burst frequency; K/ν, the average burst size; and σ/δ, the normalized standard deviation for burst frequency. This integral can be evaluated numerically by computing the distribution for a range of values for *r* and then performing a numerical integration across them.

Given this model, we fitted the parameters via a Bayesian inference approach using a Markov chain Monte Carlo (MCMC) sampling scheme implemented in the programming language Julia. This enabled us to obtain posterior distributions for each of the three parameter ratios, from which we obtained maximum *a posteriori* estimates for each parameter and 95% credible intervals, as plotted in Fig. 3C. All code relating to the modeling and parameter inference is available at https://github.com/rdbrackston/TranscriptionModels.

### Calculating extrinsic contributions to variance.

Given the model fits to each data set, we were able to calculate the extrinsic contributions to variance following an approach described elsewhere (21, 38). If n is the copy number of mRNA drawn from the compound distribution *q*(*n*/λ), where λ is the variable burst frequency, then the total variance of *n*, Var(*n*), may be determined as follows:

Var(n)=E[Var(n|λ)]+Var[E(n/λ)].

The first term is the average variance of the mRNA copy number distribution, where the average is over the distribution of values for λ. This term gives the contribution of intrinsic noise, as it is essentially a weighted sum of the variation that arises for a fixed λ. The second term is the variance of the mean copy number, where the variance is again evaluated over the distribution of values for λ. This quantifies the contribution of the extrinsic noise, since it is a measure of the variation in the copy number directly resulting from the variation in λ. We can calculate each of these terms numerically given the model parameters, thereby calculating the fractional extrinsic contribution to the total variance as follows:

$$e=\frac{\text{Var}\left[\text{E}\left[\text{n}|\lambda \right]\right]}{\text{Var}\left[\text{n}\right]}.$$

In practice, the MCMC scheme yields a joint distribution over the three parameters, λ/δ, *K*/ν, and σ/δ. In order to accurately assess a best estimate of the extrinsic contribution as well as confidence intervals, we evaluated the contribution *e* for 4,000 parameter triplets sampled from the chain. From this distribution, we calculated a maximum *a posteriori* estimate and 68% credible intervals.

10.1128/msystems.00596-22.3 FIG S3 Expression of the *nifLA* operon at high, low, and zero levels for quantification of mRNA. (A and B) DAPI staining (A) and *nifLA* mRNA-FISH probe signal (B). (i) Δ*glnB* strain grown for 3 to 4 h with 0.5 mM ammonia; (ii) M5a1 WT strain grown for 3 to 4 h with 0.5 mM ammonia; (iii) Δ*nifLA* strain grown for 4 to 6 h with 20 mM ammonia. Download FIG S3, TIF file, 2.6 MB. Copyright © 2022 Bashir et al. 2022 Bashir et al. https://creativecommons.org/licenses/by/4.0/ This content is distributed under the terms of the Creative Commons Attribution 4.0 International license.

10.1128/msystems.00596-22.4 FIG S4 Expression of the *glnK*-*amtB* operon at high, low, and zero levels for quantification of mRNA. (A and B) DAPI staining (A) and *glnK*-*amtB* mRNA-FISH probe signal (B). (i) M5a1 WT strain grown for 6 to 8 h with 0.5 mM ammonia; (ii) Δ*glnK* strain grown for 4 to 5 h with 10 mM ammonia; (iii) Δ*nifLA* strain grown for 4 to 5 h with 10 mM ammonia. Download FIG S4, TIF file, 2.9 MB. Copyright © 2022 Bashir et al. 2022 Bashir et al. https://creativecommons.org/licenses/by/4.0/ This content is distributed under the terms of the Creative Commons Attribution 4.0 International license.

## References

[1] Elowitz MB, Levine AJ, Siggia ED, Swain PS. 2002. Stochastic gene expression in a single cell. Science 297:1183–1186. doi:10.1126/science.1070919. 12183631

[2] Engl C. 2019. Noise in bacterial gene expression. Biochem Soc Trans 47:209–217. doi:10.1042/BST20180500. 30578346

[3] Cai L, Friedman N, Xie XS. 2006. Stochastic protein expression in individual cells at the single molecule level. Nature 440:358–362. doi:10.1038/nature04599. 16541077

[4] Kiviet DJ, Nghe P, Walker N, Boulineau S, Sunderlikova V, Tans SJ. 2014. Stochasticity of metabolism and growth at the single-cell level. Nature 514:376–379. doi:10.1038/nature13582. 25186725

[5] Jones D, Elf J. 2018. Bursting onto the scene? Exploring stochastic mRNA production in bacteria. Curr Opin Microbiol 45:124–130. doi:10.1016/j.mib.2018.04.001. 29705632

[6] Golding I, Paulsson J, Zawilski SM, Cox EC. 2005. Real-time kinetics of gene activity in individual bacteria. Cell 123:1025–1036. doi:10.1016/j.cell.2005.09.031. 16360033

[7] Suter DM, Molina N, Gatfield D, Schneider K, Schibler U, Naef F. 2011. Mammalian genes are transcribed with widely different bursting kinetics. Science 332:472–474. doi:10.1126/science.1198817. 21415320

[8] Larson DR, Fritzsch C, Sun L, Meng X, Lawrence DS, Singer RH. 2013. Direct observation of frequency modulated transcription in single cells using light activation. Elife 2:e00750. doi:10.7554/eLife.00750. 24069527 PMC3780543

[9] Swain PS, Elowitz MB, Siggia ED. 2002. Intrinsic and extrinsic contributions to stochasticity in gene expression. Proc Natl Acad Sci USA 99:12795–12800. doi:10.1073/pnas.162041399. 12237400 PMC130539

[10] Raser JM, O'Shea EK. 2004. Control of stochasticity in eukaryotic gene expression. Science 304:1811–1814. doi:10.1126/science.1098641. 15166317 PMC1410811

[11] Gasch AP, Yu FB, Hose J, Escalante LE, Place M, Bacher R, Kanbar J, Ciobanu D, Sandor L, Grigoriev IV, Kendziorski C, Quake SR, McClean MN. 2017. Single-cell RNA sequencing reveals intrinsic and extrinsic regulatory heterogeneity in yeast responding to stress. PLoS Biol 15:e2004050. doi:10.1371/journal.pbio.2004050. 29240790 PMC5746276

[12] Ko MS. 1991. A stochastic model for gene induction. J Theor Biol 153:181–194. doi:10.1016/s0022-5193(05)80421-7. 1787735

[13] Peccoud J, Ycart B. 1995. Markovian modelling of gene product synthesis. Theor Popul Biol 48:222–234. doi:10.1006/tpbi.1995.1027.

[14] Raj A, Peskin CS, Tranchina D, Vargas DY, Tyagi S. 2006. Stochastic mRNA synthesis in mammalian cells. PLoS Biol 4:e309. doi:10.1371/journal.pbio.0040309. 17048983 PMC1563489

[15] Iyer-Biswas S, Hayot F, Jayaprakash C. 2009. Stochasticity of gene products from transcriptional pulsing. Phys Rev E Stat Nonlin Soft Matter Phys 79:031911. doi:10.1103/PhysRevE.79.031911. 19391975

[16] Schreiber F, Littmann S, Lavik G, Escrig S, Meibom A, Kuypers MM, Ackermann M. 2016. Phenotypic heterogeneity driven by nutrient limitation promotes growth in fluctuating environments. Nat Microbiol 1:16055. doi:10.1038/nmicrobiol.2016.55. 27572840

[17] Munsky B, Fox Z, Neuert G. 2015. Integrating single-molecule experiments and discrete stochastic models to understand heterogeneous gene transcription dynamics. Methods 85:12–21. doi:10.1016/j.ymeth.2015.06.009. 26079925 PMC4537808

[18] Sepulveda LA, Xu H, Zhang J, Wang M, Golding I. 2016. Measurement of gene regulation in individual cells reveals rapid switching between promoter states. Science 351:1218–1222. doi:10.1126/science.aad0635. 26965629 PMC4806797

[19] Larsson AJM, Johnsson P, Hagemann-Jensen M, Hartmanis L, Faridani OR, Reinius B, Segerstolpe A, Rivera CM, Ren B, Sandberg R. 2019. Genomic encoding of transcriptional burst kinetics. Nature 565:251–254. doi:10.1038/s41586-018-0836-1. 30602787 PMC7610481

[20] Lenive O, Kirk PDW, Stumpf MPH. 2016. Inferring extrinsic noise from single-cell gene expression data using approximate Bayesian computation. BMC Syst Biol 10:81. doi:10.1186/s12918-016-0324-x. 27549182 PMC4994381

[21] Sherman MS, Lorenz K, Lanier MH, Cohen BA. 2015. Cell-to-cell variability in the propensity to transcribe explains correlated fluctuations in gene expression. Cell Syst 1:315–325. doi:10.1016/j.cels.2015.10.011. 26623441 PMC4662655

[22] Ham L, Brackston RD, Stumpf MPH. 2020. Extrinsic noise and heavy-tailed laws in gene expression. Phys Rev Lett 124:108101. doi:10.1103/PhysRevLett.124.108101. 32216388

[23] Dixon R, Kahn D. 2004. Genetic regulation of biological nitrogen fixation. Nat Rev Microbiol 2:621–631. doi:10.1038/nrmicro954. 15263897

[24] Schumacher J, Joly N, Rappas M, Zhang X, Buck M. 2006. Structures and organisation of AAA+ enhancer binding proteins in transcriptional activation. J Struct Biol 156:190–199. doi:10.1016/j.jsb.2006.01.006. 16531068

[25] Little R, Colombo V, Leech A, Dixon R. 2002. Direct interaction of the NifL regulatory protein with the GlnK signal transducer enables the Azotobacter vinelandii NifL-NifA regulatory system to respond to conditions replete for nitrogen. J Biol Chem 277:15472–15481. doi:10.1074/jbc.M112262200. 11856746

[26] Stips J, Thummer R, Neumann M, Schmitz RA. 2004. GlnK effects complex formation between NifA and NifL in Klebsiella pneumoniae. Eur J Biochem 271:3379–3388. doi:10.1111/j.1432-1033.2004.04272.x. 15291815

[27] Schumacher J, Behrends V, Pan Z, Brown DR, Heydenreich F, Lewis MR, Bennett MH, Razzaghi B, Komorowski M, Barahona M, Stumpf MP, Wigneshweraraj S, Bundy JG, Buck M. 2013. Nitrogen and carbon status are integrated at the transcriptional level by the nitrogen regulator NtrC in vivo. mBio 4:e00881-13. doi:10.1128/mBio.00881-13. 24255125 PMC3870243

[28] He L, Soupene E, Ninfa A, Kustu S. 1998. Physiological role for the GlnK protein of enteric bacteria: relief of NifL inhibition under nitrogen-limiting conditions. J Bacteriol 180:6661–6667. doi:10.1128/JB.180.24.6661-6667.1998. 9852012 PMC107771

[29] Dilworth MJ. 1966. Acetylene reduction by nitrogen-fixing preparations from Clostridium pasteurianum. Biochim Biophys Acta 127:285–294. doi:10.1016/0304-4165(66)90383-7. 5964974

[30] Waite CJ, Lindstrom Battle A, Bennett MH, Carey MR, Hong CK, Kotta-Loizou I, Buck M, Schumacher J. 2021. Resource allocation during the transition to diazotrophy in Klebsiella oxytoca. Front Microbiol 12:718487. doi:10.3389/fmicb.2021.718487. 34434180 PMC8381380

[31] Skinner SO, Sepulveda LA, Xu H, Golding I. 2013. Measuring mRNA copy number in individual Escherichia coli cells using single-molecule fluorescent in situ hybridization. Nat Protoc 8:1100–1113. doi:10.1038/nprot.2013.066. 23680982 PMC4029592

[32] Mc Mahon SS, Sim A, Filippi S, Johnson R, Liepe J, Smith D, Stumpf MP. 2014. Information theory and signal transduction systems: from molecular information processing to network inference. Semin Cell Dev Biol 35:98–108. doi:10.1016/j.semcdb.2014.06.011. 24953199

[33] Mc Mahon SS, Lenive O, Filippi S, Stumpf MP. 2015. Information processing by simple molecular motifs and susceptibility to noise. J R Soc Interface 12:0597. doi:10.1098/rsif.2015.0597. 26333812 PMC4614471

[34] Jones DL, Brewster RC, Phillips R. 2014. Promoter architecture dictates cell-to-cell variability in gene expression. Science 346:1533–1536. doi:10.1126/science.1255301. 25525251 PMC4388425

[35] Chong S, Chen C, Ge H, Xie XS. 2014. Mechanism of transcriptional bursting in bacteria. Cell 158:314–326. doi:10.1016/j.cell.2014.05.038. 25036631 PMC4105854

[36] Sevier SA, Kessler DA, Levine H. 2016. Mechanical bounds to transcriptional noise. Proc Natl Acad Sci USA 113:13983–13988. doi:10.1073/pnas.1612651113. 27911801 PMC5150389

[37] Paulsson J, Ehrenberg M. 2000. Random signal fluctuations can reduce random fluctuations in regulated components of chemical regulatory networks. Phys Rev Lett 84:5447–5450. doi:10.1103/PhysRevLett.84.5447. 10990965

[38] Hilfinger A, Paulsson J. 2011. Separating intrinsic from extrinsic fluctuations in dynamic biological systems. Proc Natl Acad Sci USA 108:12167–12172. doi:10.1073/pnas.1018832108. 21730172 PMC3141918

[39] Arcondeguy T, van Heeswijk WC, Merrick M. 1999. Studies on the roles of GlnK and GlnB in regulating Klebsiella pneumoniae NifL-dependent nitrogen control. FEMS Microbiol Lett 180:263–270. doi:10.1111/j.1574-6968.1999.tb08805.x. 10556721

[40] Jack R, De Zamaroczy M, Merrick M. 1999. The signal transduction protein GlnK is required for NifL-dependent nitrogen control of nif gene expression in Klebsiella pneumoniae. J Bacteriol 181:1156–1162. doi:10.1128/JB.181.4.1156-1162.1999. 9973341 PMC93492

[41] Reyes-Ramirez F, Little R, Dixon R. 2001. Role of Escherichia coli nitrogen regulatory genes in the nitrogen response of the Azotobacter vinelandii NifL-NifA complex. J Bacteriol 183:3076–3082. doi:10.1128/JB.183.10.3076-3082.2001. 11325935 PMC95207

[42] Ehira S, Miyazaki S. 2015. Regulation of genes involved in heterocyst differentiation in the cyanobacterium Anabaena sp. strain PCC 7120 by a group 2 sigma factor SigC. Life (Basel) 5:587–603. doi:10.3390/life5010587. 25692906 PMC4390870

[43] Taniguchi Y, Choi PJ, Li GW, Chen H, Babu M, Hearn J, Emili A, Xie XS. 2010. Quantifying E. coli proteome and transcriptome with single-molecule sensitivity in single cells. Science 329:533–538. doi:10.1126/science.1188308. 20671182 PMC2922915

[44] Engl C, Jovanovic G, Brackston RD, Kotta-Loizou I, Buck M. 2020. The route to transcription initiation determines the mode of transcriptional bursting in E. coli. Nat Commun 11:2422. doi:10.1038/s41467-020-16367-6. 32415118 PMC7229158

[45] So LH, Ghosh A, Zong C, Sepulveda LA, Segev R, Golding I. 2011. General properties of transcriptional time series in Escherichia coli. Nat Genet 43:554–560. doi:10.1038/ng.821. 21532574 PMC3102781

[46] Friedman LJ, Mumm JP, Gelles J. 2013. RNA polymerase approaches its promoter without long-range sliding along DNA. Proc Natl Acad Sci USA 110:9740–9745. doi:10.1073/pnas.1300221110. 23720315 PMC3683791

[47] Lestas I, Vinnicombe G, Paulsson J. 2010. Fundamental limits on the suppression of molecular fluctuations. Nature 467:174–178. doi:10.1038/nature09333. 20829788 PMC2996232

[48] Yan J, Hilfinger A, Vinnicombe G, Paulsson J. 2019. Kinetic uncertainty relations for the control of stochastic reaction networks. Phys Rev Lett 123:108101. doi:10.1103/PhysRevLett.123.108101. 31573304

[49] Carey JN, Mettert EL, Roggiani M, Myers KS, Kiley PJ, Goulian M. 2018. Regulated stochasticity in a bacterial signaling network permits tolerance to a rapid environmental change. Cell 175:1989–1990. doi:10.1016/j.cell.2018.11.051. 30550792 PMC6349375

[50] Patange O, Schwall C, Jones M, Villava C, Griffith DA, Phillips A, Locke JCW. 2018. Escherichia coli can survive stress by noisy growth modulation. Nat Commun 9:5333. doi:10.1038/s41467-018-07702-z. 30559445 PMC6297224

[51] Flemming HC, Wuertz S. 2019. Bacteria and archaea on Earth and their abundance in biofilms. Nat Rev Microbiol 17:247–260. doi:10.1038/s41579-019-0158-9. 30760902

[52] Jones K, Bradshaw SB. 1997. Synergism in biofilm formation between Salmonella enteritidis and a nitrogen-fixing strain of Klebsiella pneumoniae. J Appl Microbiol 82:663–668. doi:10.1111/j.1365-2672.1997.tb03600.x. 9172411

[53] Wang D, Xu A, Elmerich C, Ma LZ. 2017. Biofilm formation enables free-living nitrogen-fixing rhizobacteria to fix nitrogen under aerobic conditions. ISME J 11:1602–1613. doi:10.1038/ismej.2017.30. 28338674 PMC5520150

[54] Gasperotti A, Brameyer S, Fabiani F, Jung K. 2020. Phenotypic heterogeneity of microbial populations under nutrient limitation. Curr Opin Biotechnol 62:160–167. doi:10.1016/j.copbio.2019.09.016. 31698311

[55] Delvigne F, Zune Q, Lara AR, Al-Soud W, Sorensen SJ. 2014. Metabolic variability in bioprocessing: implications of microbial phenotypic heterogeneity. Trends Biotechnol 32:608–616. doi:10.1016/j.tibtech.2014.10.002. 25457387

[56] Yu Z, Li S, Li Y, Jiang Z, Zhou J, An Q. 2018. Complete genome sequence of N2-fixing model strain Klebsiella sp. nov. M5al, which produces plant cell wall-degrading enzymes and siderophores. Biotechnol Rep (Amst) 17:6–9. doi:10.1016/j.btre.2017.11.006. 29234606 PMC5723360

[57] Cannon FC, Dixon RA, Postgate JR, Primrose SB. 1974. Chromosomal integration of Klebsiella nitrogen fixation genes in Escherichia coli. J Gen Microbiol 80:227–239. doi:10.1099/00221287-80-1-227. 4595005

[58] Shah VK, Brill WJ. 1973. Nitrogenase. IV. Simple method of purification to homogeneity of nitrogenase components from Azotobacter vinelandii. Biochim Biophys Acta 305:445–454. doi:10.1016/0005-2728(73)90190-4. 4354875

