# Supplementary figures and images for: Molecular Origins of Transcriptional Heterogeneity in Diazotrophic Klebsiella oxytoca
Source: mSystems. 2022 Sep 8;7(5):e00596-22. doi: 10.1128/msystems.00596-22 (PMC9600154; doi:10.1128/msystems.00596-22)

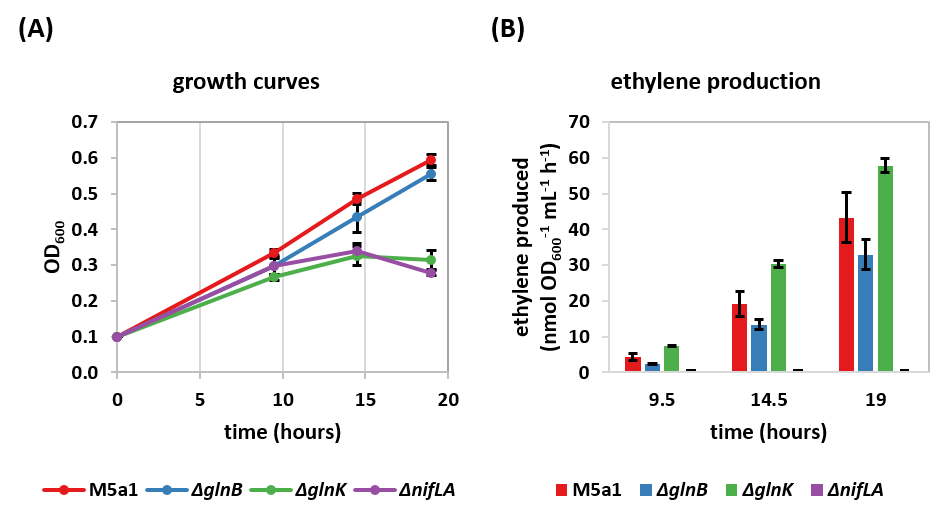

Supplement: FIG S1 [file msystems.00596-22-s0001.tif]

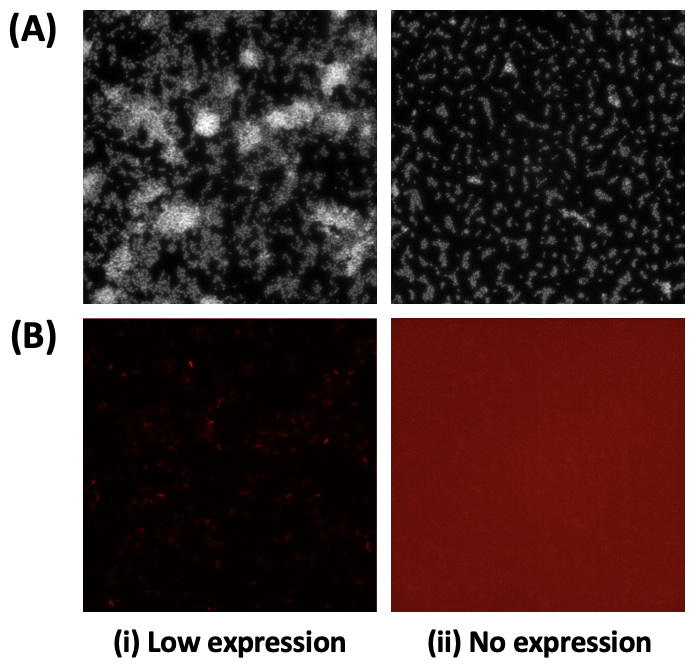

Supplement: FIG S2 [file msystems.00596-22-s0002.tif]

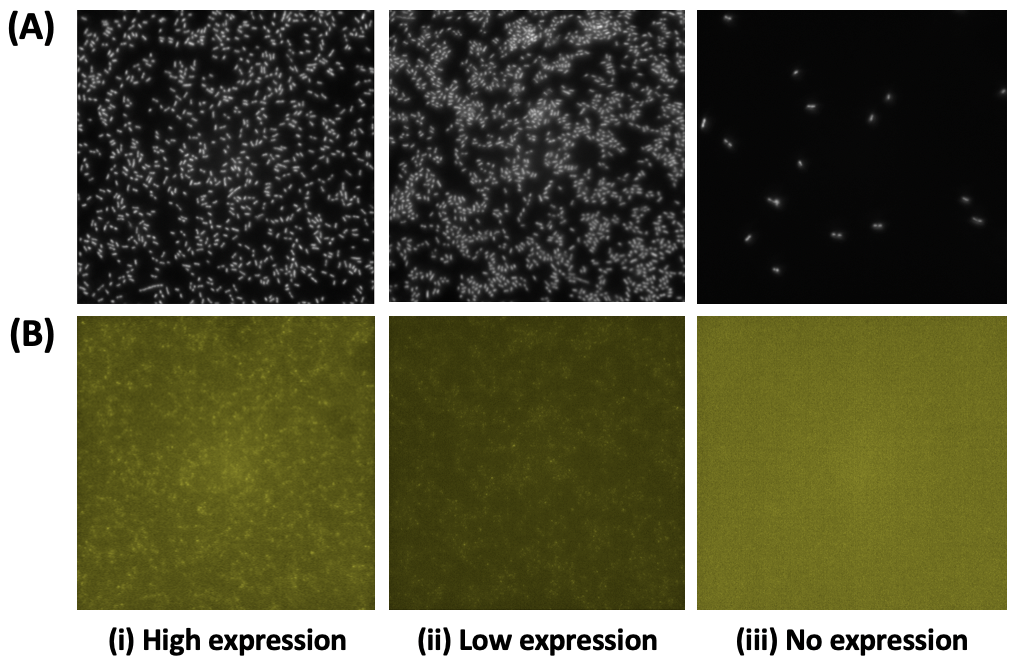

Supplement: FIG S3 [file msystems.00596-22-s0003.tif]

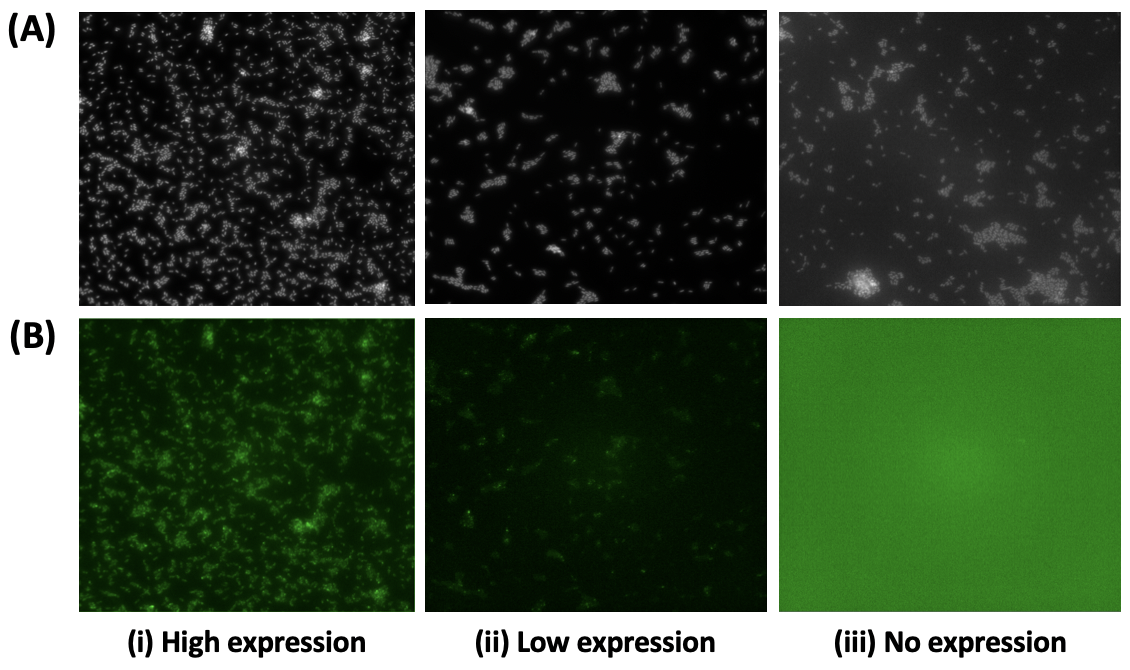

Supplement: FIG S4 [file msystems.00596-22-s0004.tif]
