# Supplementary material for: Molecular Origins of Transcriptional Heterogeneity in Diazotrophic Klebsiella oxytoca
Source: mSystems. 2022 Sep 8;7(5):e00596-22. doi: 10.1128/msystems.00596-22 (PMC9600154; doi:10.1128/msystems.00596-22)
Supplement: TABLE S1 [file msystems.00596-22-s0005.docx]

**Table S1.** Names and sequences of the oligonucleotide primers used to construct knockout mutants.

| **Name** | **Sequence (5'-3')^1^** |
| --- | --- |
| *nifLA*_mutF | ccgcggcgtccctgtcacggtgtcggacaaattgtcataactgcgacacaggagtttgcgGTGTAGGCTGGAGCTGCTTC |
| *nifLA*_mutR | acaaattgtcgcaattccgccgcgctggcgacaatgtcctgaatctcacataaggcttcaTCCTCCTTAGTTCCTATTCCG |
| *glnK*_mutF | ctgcggccattaccgaattctgactggaggggacttatgaagctggttaccgtggtaatcGTGTAGGCTGGAGCTGCTTC |
| *glnK*_mutR | tgttttcattgttgctatcttcattgtttcgttccccatcactgtgtggtctgaaagttaTCCTCCTTAGTTCCTATTCCG |
| *glnB*_mutF | taggatgacgcaagttatgatacggttagcaaccggttacgcgactaagacaggaacaccGTGTAGGCTGGAGCTGCTTC |
| *glnB*_mutR | cggggctacaaatctggatggaattgaagttcttaagccatcatccgtcttttaagcttaTCCTCCTTAGTTCCTATTCCG |
| *amtB*_mutF | ggcgaatctgacgaagcggcactgtaactttcagaccacacagtgatggggaacgaaacaGTGTAGGCTGGAGCTGCTTC |
| *amtB*_mutR | taaccatagaaaagcggagccaggctccgctttttgtcgattaaagataacgattaatcaTCCTCCTTAGTTCCTATTCCG |

^1^lower case letters indicate sequences homologous to the flanking regions of the target gene; upper case letters indicate sequences homologous to the kanamycin cassette
