# Supplementary material for: Molecular Origins of Transcriptional Heterogeneity in Diazotrophic Klebsiella oxytoca
Source: mSystems. 2022 Sep 8;7(5):e00596-22. doi: 10.1128/msystems.00596-22 (PMC9600154; doi:10.1128/msystems.00596-22)
Supplement: TABLE S2 [file msystems.00596-22-s0006.docx]

**Table S2.** Names and sequences of the oligonucleotide probes used for mRNA fluorescence *in situ* hybridisation.

| ***nifHDK* (96 probes) Quasar^1^** | | ***nifLA* (48 probes) 6-TAMRA** | ***glnKamtB* (48 probes) 6-TAMRA** |
| --- | --- | --- | --- |
| ccgtaaatagcgcattgacg | atatagagcagcaccttacg | agcatcatattcagggtcat | tttgattaccacggtaacca |
| catctccataatggtgttct | tcataggcgccaataacgtg | ccattgtaaaaaacagcccc | gcacatcttccagtttgaat |
| gaaagttgatcgccgtgatc | tcgttatgggcaaactcgta | atcggtgagggaaatcgcta | cctgaatacccattgaagat |
| cacgaaatcgagatcgtcct | cgtcatcgaacagcagcgtg | tggcgtagataatattcgcc | ctttaacttcagtgaccgtc |
| cagacgatgtagatctcctg | ttgatgccggagccgataag | gcgggttttgattgagcaat | aattgacgctgtattcggcg |
| tccctttggagatattgttg | gcccattttctggaagatat | cacatctcctggtagatctc | cgcgacatcaatcttcactt |
| ccggatttggcgtatttaac | aatagtcccacgagtgcatc | ggcgctgattaattagctga | gatgacatcgacgacttcgt |
| gggcaataatcagttcgtct | aatggcgaaaccatcgtagc | gcgtgatatcgatatctacc | cgacaaagattttgccgtcg |
| cgtactcgataaccgtcatg | ttcagggtcatatccatatc | ggtatagctgacgctgatat | cggtacggatgcgaatcacg |
| tggtgttgttgacgatcttc | gacagtgagtgggcaatcac | ttagcgtcatatgattgcgc | tcactgtgtggtctgaaagt |
| atcataattgtcctgtgctc | gccacaaatcggtgaacaga | atgttattgagcaccgcttc | gctatcttcattgtttcgtt |
| agagataatgcacttgccga | atcaatcgtttggctcatgg | tttgtaggcgagattatcca | cggaagaagagccaacgctc |
| gaaatatgggccatatcctt | tcgaatagcggataacagct | gtcatttgctgtttcagacg | aaagcgttgtcggctttatc |
| atcagaggtgaagttcagcg | aacagctcctggtattcatc | aattaagctgaatcagcgcc | cagcgcggtgcaaatcatca |
| tcttcaatcagcttgctgag | aattcagcgcttcatactcg | ctttccagctcaagagaagg | cgatgccaggaatcgacata |
| tgatatcatcaccgatcagc | aaatcccagcgagcaaagca | aaaaaagggctgaagcggcc | agaacgtttttaccgcggat |
| gatgatcgccacatcgtaag | aataggtgcgaaagtaggcc | gtcgagccataaactcaagc | cgatcacctgagtcagcatg |
| aggttcagcttgacgaatgg | gatcggctctttgaaatggc | ttcggcgtaaagctcgatct | cacgcagaccagcgcaaaag |
| agttcatcgaacggtagcag | tcatcgagtcggagacgcag | agatacaaagagagccagcc | agcgtgtagccataaaccac |
| acggaatctgatgtttctcc | ccaggttcatattgttgttg | cagggtattaaagagcggga | aagttaccgaagaagctgcc |
| ccgaagaagttgtactccat | aatgatctccggtttgtaca | tttatggatcattgctcacc | aatgtttttcagcagtaccc |
| cgcgaatggtatcgtcgaac | aggtcatcgccgataacttc | aaacgtctgacggtggtgtc | tgatagaagcttcccatcag |
| atatttggcgataatcgccg | cttttttagcgttggcgata | ggtaaactgctgggagagat | ctggaaggcaacgtggatgt |
| atatagagcagcaccttacg | cgatgctgctgtcgacgaag | gttatgtagcacgctcagaa | agaccaacggtaatacaggc |
| tcgttatgggcaaactcgta | ccgataaagcttggcgtatg | ctgtcgtacaggcaaatcat | cgagaagcgaatgcgttccg |
| aatggcgaaaccatcgtagc | ttcaaacatgttatcccagc | ggtagcgaatttgcgtactg | tcatccataccacgacgaaa |
| ttcagggtcatatccatatc | cagtgaaggttttggcgaag | cagcacggtaccgactaatc | cgcaatcggcacataagaga |
| gccacaaatcggtgaacaga | cagattgagcttcggcaatt | tcgagaaaacgctggtcgtc | gttgatgtgaaccaccgtac |
| atcaatcgtttggctcatgg | gagataggtttcaaagccgg | atagtcgtacaggctcagac | tttgcccatcatataggcac |
| ggtattcatcctgttcgaat | ttccatcatccgctttaata | cagcggaacggcgataaacg | gtttgaacgcttctttacca |
| aattcagcgcttcatactcg | tcgagaacttccgacggatc | tggcgacggtttcgagaaag | aataccatcggcaggttatg |
| aataggtgcgaaagtaggcc | ccggaatacatccgatagtg | caggatcatcaggcgaatcg | aaccagccaacgtagaggat |
| gcgatcggctctttgaaatg | acttttttgctcttcagcag | catattttccaggccgaaac | tggctgaaccggcgttaaag |
| aatgatctccggtttgtaca | tcatcagcagttcatcggtg | tatccataatctgccgcatc | cagaccggcaatttcgttcg |
| tcgacgaagccatctttttt | gagtcgagcatcatgtcaac | cagcgggaaacctgacgaat | ccaccacggtattaacgaac |
| ccgataaagcttggcgtatg | cgtacaggccaaacttcttg | gaattatggtggatggcgtt | gaaggtccacgccagaatag |
| tggcgaagccttcaaacatg | cagcgtttgttggcgttatg | ccgcgcagttaaatttgacg | cagcagagatggtttaccgc |
| cagattgagcttcggcaatt | agcattttgttcatcgcttt | tctcatgaccaaacagctcg | caatcgcacctgaacaggcg |
| gagataggtttcaaagccgg | taaacacttcgctatcgcgc | tcatcgaggaataaggtgcc | cagcgataccgacaatcaga |
| ttccatcatccgctttaata | aagtgccacaaatcgcagtt | tgaaacgaggcgctgctttc | cagcgtttcagtacggtgac |
| ccggaatacatccgatagtg | acgggtgaacatcagcgaac | gcagaatacgcagtagctta | aaacacatcgcatgggtcat |
| gagtcgagcatcatgtcaac | ggagttgccgatcataaagt | gcgataatgcgcacgttgac | caggatgcagcctacgatac |
| ttttgttcatcgctttttgc | gtatcgcgctggataaactt | tagtatagatcctcgcggaa | agacgtcgcggcgaagatac |
| gccacaaatcgcagttgata | ataagcggcacttcaaaggc | cgataggcattacgttcagg | agctgtaccagaacctgatg |
| gatcataaagtccggctgac | tcgaacagcggaaagccgag | tttttcgcaccagaaagtgc | cagacgatggtgataccgat |
| ctggataaacttgccgtagg | cgtcgtcacaatgttcatcg | aacgttcgagacagttttcc | aaccaatgaaggcgacgacg |
| cggataagcggcacttcaaa | tggtatcgctatccagtttc | ggttgaacagaatcacgtcc | aacggtcatatcggcaactt |
| cgtcgtcacaatgttcatcg | cggacgagatcgaagctgta | aatctgaatgcgatacgcca | ttcacgctcttgatcttccg |

^1^two sets of 48 probes each were used for detection of *nifHDK* mRNA
